# Advancing Elder Mistreatment Research Through Theory: Exploring Mechanisms and Developing Models

**DOI:** 10.1093/geroni/igaf122.1422

**Published:** 2025-12-31

**Authors:** Kelly Marnfeldt, Susanna Mage, Kathleen Wilber

## Abstract

Elder mistreatment (EM) is a critical yet under-addressed public health issue, with family caregivers among the most likely to perpetrate mistreatment. This symposium underscores the importance of strong theoretical frameworks in advancing EM research and developing effective prevention strategies. Historically described as “data rich and theory poor,” the field must strengthen its theoretical foundations to drive more robust, evidence-based approaches. Dr. Wilber will serve as the discussant, guiding a conversation on integrating theory to enhance research and practice. Dr. Meyer will begin by describing the iterative integration of Resourcefulness and Quality of Life Theory into the KINDER intervention to establish a theory-based mechanism of change. Next, Dr. Marnfeldt will present a case analysis of the COACH program, emphasizing the need to move beyond identifying risk factors to understanding why mistreatment occurs and how interventions work. Dr. Avent will then discuss findings from the Better Together Couples’ Study, applying the web of violence framework and life course theory to explore how past abuse shapes caregivers’ recognition of mistreatment and their caregiving experiences, strengthening support for trauma-informed, lifespan-focused interventions. Following, Dr. Rosen will introduce a theoretical model outlining multiple pathways through which EM may increase Medicaid expenditures, identifying eight intervention targets to guide research on Medicaid costs, eligibility, and transitions. Lastly, Dr. Bloemen will examine the complex ways EM affects families, illustrating how family systems and resiliency theory can enhance acute care responses for victims. Collectively, these presentations advance EM research by strengthening theoretical foundations and informing more effective prevention strategies. Abuse, Neglect and Exploitation of Older Persons Interest Group Sponsored Symposium

